# Psychotropic prescribing after hospital discharge in survivors of critical illness, a retrospective cohort study (2012–2019)

**DOI:** 10.1177/17511437231223470

**Published:** 2024-01-19

**Authors:** Elizabeth T Mansi, Christopher T Rentsch, Richard S Bourne, Bruce Guthrie, Nazir I Lone

**Affiliations:** 1 Usher Institute, University of Edinburgh, Edinburgh, UK; 2 Faculty of Epidemiology and Population Health, London School of Hygiene & Tropical Medicine, London, UK; 3 Department of Internal Medicine, Yale School of Medicine, New Haven, CT, USA; 4 Departments of Pharmacy and Critical Care, Sheffield Teaching Hospitals NHS Foundation Trust, Sheffield, UK; 5 Division of Pharmacy and Optometry, School of Health Sciences, Faculty of Biology, Medicine and Health, The University of Manchester, Manchester, UK; 6 Advanced Care Research Centre, University of Edinburgh, Edinburgh, UK; 7 Department of Anaesthesia, Critical Care, and Pain Medicine, School of Clinical Sciences, University of Edinburgh, Edinburgh, UK

**Keywords:** Psychotropic drugs, critical care, critical Illness, post-intensive care syndrome, mental disorders

## Abstract

**Background:**

Many people survive critical illness with the burden of new or worsened mental health issues and sleep disturbances. We examined the frequency of psychotropic prescribing after critical illness, comparing critical care to non-critical care hospitalised survivors, and whether this varied in important subgroups.

**Methods:**

This retrospective cohort study included 23,340 critical care and 367,185 non-critical care hospitalised adults from 2012 through 2019 in Lothian, Scotland, who survived to discharge.

**Results:**

One-third of critical care survivors (32%; 7527/23,340) received a psychotropic prescription within 90 days after hospital discharge (25% antidepressants; 14% anxiolytics/hypnotics; 4% antipsychotics/mania medicines). In contrast, 15% (54,589/367,185) of non-critical care survivors received a psychotropic prescription (12% antidepressants; 5% anxiolytics/hypnotics; 2% antipsychotics/mania medicines). Among patients without psychotropic prescriptions within 180 days prior to hospitalisation, after hospital discharge, the critical care group had a higher incidence of psychotropic prescription (10.3%; 1610/15,609) compared with the non-critical care group (3.2%; 9743/307,429); unadjusted hazard ratio (HR) 3.39, 95% CI: 3.22–3.57. After adjustment for potential confounders, the risk remained elevated (adjusted HR 2.03, 95% CI: 1.91–2.16), persisted later in follow-up (90–365 days; adjusted HR 1.38, 95% CI: 1.30–1.46), and was more pronounced in those without recorded comorbidities (adjusted HR 3.49, 95% CI: 3.22–3.78).

**Conclusions:**

Critical care survivors have a higher risk of receiving psychotropic prescriptions than hospitalised patients, with a significant proportion receiving benzodiazepines and other hypnotics. Future research should focus on the requirement for and safety of psychotropic medicines in survivors of critical illness, to help guide policy for clinical practice.

## Introduction

Every year, over 160,000 people survive critical illness in the UK.^1,2^ Global meta-analyses on mental health disorders after critical illness have been published in recent years showing that clinically important depression, anxiety, and post-traumatic stress disorder (PTSD) symptoms occur within a year of follow-up in one-third,^3^ one-third^4^, and one-fifth^5^ of critical care survivors, respectively. In the UK, this was demonstrated in a survey of critical illness survivors, where over half of the 4943 respondents reported significant symptoms of anxiety, depression, and/or PTSD at three or 12 months following intensive care unit (ICU) discharge.^6^ Sleep disturbances are common as well, and can exacerbate symptoms.^7,8^ Reportedly, over half of critical care survivors had sleep disturbances within the first month after hospital discharge which was frequently associated with post-discharge psychological comorbidity and decreased quality of life.^9^ The incidence of pharmacologic treatment for these conditions in survivors of critical illness in the UK is currently not well-described.

Use of psychotropic medicines has increased internationally over recent decades, but few studies report psychotropic prescribing trends in survivors of critical illness or most lack comparator groups. Critical care patients are likely to have a higher level of exposure to psychotropics during their acute illness, for example, antipsychotics for management of delirium. Survivors of critical illness may be prescribed psychotropics after hospital discharge as an after effect of exposure to these medicines whilst acutely ill or due to the combined effects of mental health issues or sleep disturbances secondary to their critical illness. We aimed to examine the frequency of new and continued psychotropic prescribing after critical illness, comparing critical care survivors to non-critical care hospitalised survivors in the Lothian region of Scotland, and whether this varied in important subgroups.

## Methods

### Study design, setting, participants

**Figure 1.**
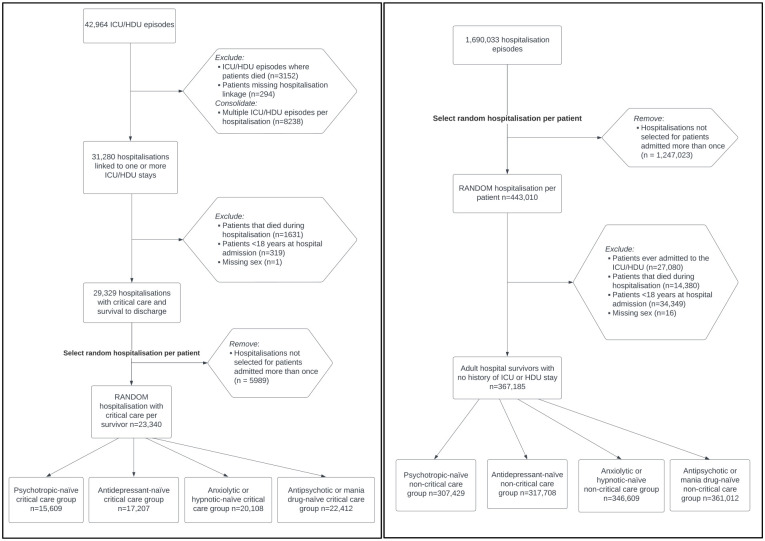
Flow diagrams of critical care group (left) and non-critical care group (right). ‘Medication-naïve’ groups include only patients that did not receive a community prescription for the specific psychotropic group within 180 days prior to index hospitalisation. Medication groups are **not** mutually exclusive. ICU: Intensive Care Unit; HDU: High Dependency Unit.

This retrospective cohort study was a secondary analysis of a larger study of patients who attended an emergency department (ED) in Lothian, Scotland from 2012 through 2019. Lothian is a mixed urban-rural area of Scotland which includes City of Edinburgh and surrounding area, with a population of approximately 897,770 as of 2018.^10^ Our study population comprised all adults (⩾18 years) admitted for inpatient hospitalisation to one of the three Lothian acute (non-mental health) hospitals between 1 January 2012 and 31 December 2019 and who survived to hospital discharge. All study patients were required to have at least one Lothian ED attendance *at any point* within the eight-year study period to be included. Patients in the critical care group were excluded from the non-critical care hospitalised group. Patients with missing data for sex (<0.01%) were excluded. For patients hospitalised more than once during the study period, one random hospitalisation event was selected so that all individuals were represented only once (Figure 1).

### Data sources and linkage

The following datasets were linked by anonymised patient identifier: Scottish Intensive Care Society Audit Group (SICSAG, critical care admissions), Prescribing Information System (PIS, community dispensed prescriptions), Scottish Morbidity Record Inpatient (SMR01, hospitalisations), Mental Health Inpatient (SMR04, psychiatric admissions), TrakCare (electronic medical records system for Lothian hospitals), and National Records of Scotland (NRS) mortality registration. Access to the data were approved by the National Health Service (NHS) Lothian/University of Edinburgh Dataloch Project Review and Advisory Committee (ref. SH2019-008), which oversees all aspects of information governance, ethics and confidentiality for studies accessing anonymised healthcare data held by the NHS Lothian. All data were analysed in a safe haven environment.

### Variables

*Outcomes*: The main outcomes of interest were receipt of community psychotropic prescription within 90 days of hospital discharge (binary), as obtained from the PIS dataset. Psychotropic prescription outcomes were categorised into four groups by British National Formulary Chapter (BNF) classification: (1) any psychotropic medicine (aggregate of the following); (2) antidepressants (BNF 4.3); (3) anxiolytics (BNF 4.1.2) or hypnotics (BNF 4.1.1); or (4) antipsychotics (BNF 4.2.1) or medications used for mania and hypomania (BNF 4.2.3). Antipsychotic depot injections (BNF Ch 4.2.2) were not included in this study, and levomepromazine was excluded from the antipsychotic outcome because in Scotland, this drug is used almost exclusively for end-of-life care. Patients may have been prescribed multiple classes of psychotropic. See Appendix A for BNF listings of psychotropic groups by generic name. For patients to be at risk for incident (new) psychotropic prescription, they were required not to have received a prescription for the medicine group of interest within 180 days prior to hospitalisation (‘naïve patients’). Secondary outcomes were late incident psychotropic prescribing from 91 to 365 days after hospital discharge in 90-day survivors. For patients to be at risk for late incident prescribing, they had to be naïve to the psychotropic group 180 days before hospitalisation and also for the 90 days after hospital discharge.

*Exposure*: The primary exposure was critical care admission during hospitalisation as measured by ICU or high-dependency unit (HDU) admission from the SICSAG. The non-critical care hospitalised comparator group served as a reference population to understand baseline rates of community psychotropic prescribing and the relative contribution made by features associated with critical care admission.

**Figure 2.**
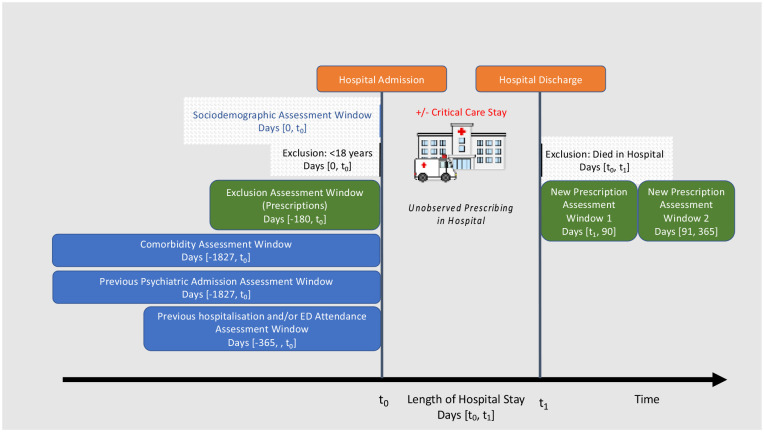
Graphical depiction of study design. ED: emergency department.

*Covariates*: Sociodemographic variables were obtained from hospital admission data and included sex, age, ethnicity, and socioeconomic status (by Scottish index of multiple deprivation (SIMD) quintiles, based on 2020 data from postcode of residence). Other variables included main condition at hospital admission, comorbidity count, previous psychiatric hospital admission, previous acute hospital admissions, and previous ED attendances. Main condition at hospital admission was categorised into 10 categories defined by International Classification of Diseases - Tenth Edition (ICD-10) codes: circulatory (I00-I99), neoplasms (C00-D49), injury or poisoning (S00-T88), digestive (K00-K95), respiratory (J00-J99), symptoms, signs and abnormal clinical and laboratory findings, not elsewhere classified (R00-R99), genitourinary (N00-N99), musculoskeletal (M00-M99), infectious (A00-B99), and all other conditions. The number of comorbidities was calculated based on a count of the Elixhauser-defined list of comorbidities^11^ using hospital discharge code information from 5 years prior to index hospitalisation and categorised into zero, one, two or more comorbidities. Previous psychiatric admission was defined as any mental health hospital admission in the 5 years prior to index hospitalisation with binary categorisation. Both previous acute hospital admissions and previous ED attendances were measured in the 1 year prior to index hospitalisation and categorised as zero, one, two or more admissions or attendances (Figure 2).

### Statistical methods

Baseline characteristics were reported using proportions, means with standard deviation, or medians with interquartile ranges. Unadjusted cumulative incidence plots for days-to-new prescription of each of the four prescription groups by critical care status were estimated using the *tidycmprsk* package in R. The associations between critical care hospitalisation and each of the four outcomes were evaluated using cause-specific Cox proportional hazard models for early (0–90 days) and late (91–365 days) psychotropic prescribing after hospital discharge with deaths during follow-up as competing events. Cause-specific hazards ratios (HR) with 95% confidence intervals (CI) were estimated. Missing data affected two covariates – ethnicity and SIMD. For ethnicity, we employed a missing category approach while those with missing SIMD data were excluded from multivariable analyses. No violations of the proportional hazard assumption were noted on visual inspection of Schoenfeld plots. All analyses were performed using R version 4.1.3.

### Effect modification

We conducted analyses to identify whether the exposure-outcome relationship varied by important subgroups, defined by age group (<60 years and 60 or older), sex, SIMD quintile, and comorbidity category, reporting *p*-values for interaction terms.

### Sensitivity analyses

We conducted three sensitivity analyses. The first sensitivity analysis excluded patients who died in the first 30 days from hospital discharge to assess if prescription patterns would be different in patients who were less likely to have been discharged for palliative care. The second sensitivity analysis excluded antidepressants prescribed at daily doses typically used for pain management in the UK rather than for treating depression, based on expert deliberation amongst the author group (ICU consultant pharmacist, ICU physician, general practitioner (GP)). These were amitriptyline ⩽25 mg, clomipramine ⩽10 mg, duloxetine (any dose), imipramine ⩽50 mg, and nortriptyline ⩽25 mg. Lastly, the third sensitivity analysis censored patients at hospital readmission. We did this because subsequent prescription may not be related to the index hospitalisation and because during hospitalisation, patients are not at risk for community prescribing.

## Results

### Patient characteristics

There were 23,340 critical care and 367,185 non-critical care hospital survivors admitted to a Lothian hospital from 2012 through 2019. The median age was 62 years (interquartile range (IQR): 48–73) in the critical care group and 56 years (IQR: 38–74) in the non-critical care group (Table 1). In the critical care group, 44% were women compared to 53% in the non-critical care group. The majority of patients with known ethnicity were white with 14.2% of the study population missing ethnicity. The most common main condition at hospitalisation in the critical care group was circulatory system diseases (20.3%) and in the non-critical care group was injury or poisoning (17.1%).

**Table 1.** Characteristics of the critical care and non-critical care hospitalised survivors in Lothian (2012–2019) in the full study population and in the subgroup of psychotropic-naïve hospitalised survivors.

		Full study population		Psychotropic-naïve patients^a^	
		Critical care *n* = 23,340 (%)	Non-critical care *n* = 367,185 (%)	Critical care *n* = 15,609 (%)	Non-critical care *n* = 307,429 (%)
Sex	Female	10,223 (43.8)	193,168 (52.6)	5924 (38.0)	154,149 (50.1)
	Male	13,117 (56.2)	174,017 (47.4)	9685 (62.0)	153,280 (49.9)
Age	Median (IQR)	62 (48–73)	56 (38–74)	64 (48–74)	56 (37–74)
Age group	18–29	1762 (7.5)	54,479 (14.8)	1338 (8.6)	48,148 (15.7)
	30–39	1893 (8.1)	43,388 (11.8)	1201 (7.7)	36,303 (11.8)
	40–49	2733 (11.7)	47,712 (13.0)	1552 (9.9)	38,369 (12.5)
	50–59	4021 (17.2)	54,201 (14.8)	2441 (15.6)	44,091 (14.3)
	60–69	5171 (22.2)	52,816 (14.4)	3468 (22.2)	44,149 (14.4)
	70–79	5069 (21.7)	54,771 (14.9)	3601 (23.1)	46,166 (15.0)
	80+	2691 (11.5)	59,818 (16.3)	2008 (12.9)	50,203 (16.3)
Ethnicity	Asian	274 (1.4)	5755 (1.8)	213 (1.6)	5194 (2.0)
	Black	116 (0.6)	1864 (0.6)	94 (0.7)	1666 (0.6)
	Mixed or other	87 (0.4)	2037 (0.6)	67 (0.5)	1792 (0.7)
	White	19,686 (97.6)	305,034 (96.9)	12,887 (97.2)	253,989 (96.7)
	Missing	3177	52,495	2348	44,788
Socioeconomic deprivation	1 (most deprived)	3713 (16.1)	54,953 (15.1)	2242 (14.5)	44,552 (14.7)
	2	5853 (25.3)	88,973 (24.5)	3664 (23.7)	72,925 (24.0)
	3	4238 (18.3)	65,883 (18.2)	2814 (18.2)	54,972 (18.1)
	4	3898 (16.9)	64,330 (17.7)	2745 (17.8)	54,713 (18.0)
	5 (least deprived)	5400 (23.4)	88,649 (24.4)	3979 (25.8)	76,383 (25.2)
	Missing	238	4397	165	3884
Year of hospital discharge	2012	2682 (11.5)	45,459 (12.4)	1805 (11.6)	38,082 (12.4)
	2013	2889 (12.4)	44,693 (12.2)	1959 (12.6)	37,599 (12.2)
	2014	3082 (13.2)	46,459 (12.7)	2106 (13.5)	39,387 (12.8)
	2015	2947 (12.6)	45,578 (12.4)	1992 (12.8)	38,120 (12.4)
	2016	2882 (12.3)	42,689 (11.6)	1920 (12.3)	35,619 (11.6)
	2017	2934 (12.6)	43,888 (12.0)	1922 (12.3)	36,579 (11.9)
	2018	2980 (12.8)	44,768 (12.2)	1961 (12.6)	37,501 (12.2)
	2019	2820 (12.1)	52,879 (14.4)	1944 (12.5)	44,542 (14.5)
	2020	124 (0.5)	772 (0.2)	74 (0.5)	654 (0.2)
Main condition at hospitalisation	Circulatory	4729 (20.3)	35,202 (9.6)	3596 (23.0)	31,132 (10.1)
	Neoplasms	4555 (19.5)	20,485 (5.6)	3157 (20.2)	16,961 (5.5)
	Injury or poisoning	3704 (15.9)	62,861 (17.1)	2210 (14.2)	53,260 (17.3)
	Digestive	3455 (14.8)	36,153 (9.8)	2336 (15.0)	30,650 (10.0)
	Respiratory	2273 (9.7)	29,280 (8.0)	1369 (8.8)	24,241 (7.9)
	Abnormal findings^b^	724 (3.1)	51,402 (14.0)	425 (2.7)	43,016 (14.0)
	Genitourinary	977 (4.2)	25,726 (7.0)	642 (4.1)	21,210 (6.9)
	Musculoskeletal	480 (2.1)	26,972 (7.3)	261 (1.7)	21,481 (7.0)
	Infectious	631 (2.7)	8660 (2.4)	409 (2.6)	7349 (2.4)
	All other conditions	1812 (7.8)	70,444 (19.2)	1204 (7.7)	58,129 (18.9)
Length of hospital stay (days)	Mean (SD)	20.2 (38.6)	5.4 (20.7)	19.5 (37.1)	5.4 (21.1)
	Median (IQR)	9 (5–19)	1 (0–4)	9 (5–18)	1 (0–3)
Number comorbidities at hospital admit	0	10,986 (47.1)	305,371 (83.2)	8136 (52.1)	269,314 (87.6)
	1	4193 (18.0)	29,285 (8.0)	2665 (17.1)	19,227 (6.3)
	2+	8161 (35.0)	32,529 (8.9)	4808 (30.8)	18,888 (6.1)
Previous psychiatric admission		780 (3.3)	4254 (1.2)	180 (1.2)	1172 (0.4)
Number hospital admissions	0	11,515 (49.3)	312,726 (85.2)	8215 (52.6)	272,786 (88.7)
	1	5607 (24.0)	34,237 (9.3)	3750 (24.0)	22,838 (7.4)
	2+	6218 (26.6)	20,222 (5.5)	3644 (23.3)	11,805 (3.8)
Number ED attendances	0	13,554 (58.1)	285,130 (77.7)	9718 (62.3)	251,186 (81.7)
	1	6001 (25.7)	57,423 (15.6)	3947 (25.3)	41,235 (13.4)
	2+	3785 (16.2)	24,632 (6.7)	1944 (12.5)	15,008 (4.9)
Died within 90 days of hospital discharge		772 (3.3)	12,705 (3.5)	472 (3.0)	10,059 (3.3)
Prescribed psychotropic drug in the 180 days prior to hospital admission^c^	Any psychotropic	7731 (33.1)	59,756 (16.3)	NA	NA
	Antidepressant	6133 (26.3)	49,477 (13.5)	NA	NA
	Anxiolytic or hypnotic	3232 (13.8)	20,576 (5.6)	NA	NA
	Antipsychotic or mania drug	928 (4.0)	6173 (1.7)	NA	NA

SD: standard deviation; IQR: interquartile range; ED: emergency department.

a Psychotropic-naïve patients are those that did not receive a community prescription for a psychotropic within 180 days prior to index hospitalisation.

b *Abnormal findings*: Abnormal laboratory or clinical findings not elsewhere specified.

c Individual psychotropic categories are not mutually exclusive. ICU: Intensive care unit or high-dependency unit; SIMD: Scottish Index of Multiple Deprivation.

### Early (0–90 day) psychotropic prescribing after hospital discharge in all patients

In the full study population, one-third (32%; 7527/23,340) of critical care and 15% of non-critical care survivors (54,589/367,185) received at least one psychotropic prescription within 90 days after hospital discharge (Table 2). Among those prescribed any psychotropic medicines, antidepressants were the most commonly prescribed drug group (critical care 76% (5734/7527) vs non-critical care 69% (37,604/54,589)), and prescriptions for more than one psychotropic group were more common in critical care survivors (critical care 27% (2055/7527) vs non-critical care 22% (12,202/54,589)) (Supplemental Figure S1).

**Table 2.** Medicines prescribed to hospitalised patients within 90 days of hospital discharge by drug class in the full study population and in the subgroup of psychotropic-naïve hospitalised survivors *(% calculated using denominator in row header)*.

Drug class	Full study population		Psychotropic-naïve patients^a^	
	Critical care *n* = 23,340 (%)	Non-critical care *n* = 367,185 (%)	Critical care *n* = 15,609 (%)	Non-critical care *n* = 307,429 (%)
No psychotropic	15,813 (67.8)	312,596 (85.1)	13,999 (89.7)	297,686 (96.8)
Any psychotropic	7527 (32.2)	54,589 (14.9)	1610 (10.3)	9743 (3.2)
Antidepressants	5734 (24.6)	44,604 (12.1)	868 (5.6)	6241 (2.0)
SSRI/SNRI	3033 (13.0)	26,285 (7.2)	337 (2.2)	2799 (0.9)
Tricyclic and related antidepressants	2086 (8.9)	13,673 (3.7)	344 (2.2)	2365 (0.8)
Other antidepressants	1473 (6.3)	10,218 (2.8)	234 (1.5)	1362 (0.4)
Anxiolytics or hypnotics	3197 (13.7)	17,276 (4.7)	873 (5.6)	3934 (1.3)
Benzodiazepines	1977 (8.5)	11,762 (3.2)	375 (2.4)	2283 (0.7)
Z-drugs	1482 (6.3)	6353 (1.7)	551 (3.5)	1768 (0.6)
Other anxiolytic or hypnotic	121 (0.5)	714 (0.2)	33 (0.2)	137 (<0.1)
Antipsychotics or drugs for mania	937 (4.0)	6453 (1.8)	114 (0.7)	842 (0.3)
First generation antipsychotics	326 (1.4)	2278 (0.6)	62 (0.4)	479 (0.2)
Second generation antipsychotics	612 (2.6)	4221 (1.2)	53 (0.3)	390 (0.1)
Drugs for mania	72 (0.3)	485 (0.1)	<10 (<0.1)	12 (<0.1)

SSRI: selective serotonin reuptake inhibitors; SNRI: serotonin-norepinephrine reuptake inhibitors. Note: Patients may have been prescribed more than one class of medication and therefore the sum of the individual drug classes may be greater than the total in the psychotropic group.

a Psychotropic-naïve patients are those that did not receive a community prescription for a psychotropic within 180 days prior to index hospitalisation.

### Psychotropic-naïve patients

Psychotropic-naïve patients are those that did not receive a community prescription for a psychotropic within 180 days prior to index hospitalisation. Of the 23,340 survivors of critical illness, there were 15,609 psychotropic-naïve (67%), 17,207 antidepressant-naïve (74%), 20,108 anxiolytic or hypnotic-naïve (86%), and 22,412 antipsychotic or mania medicine-naïve (96%) patients. Of the 367,185 non-critical care survivors, there were 307,429 psychotropic-naïve (84%), 317,708 antidepressant-naïve (87%), 346,609 anxiolytic or hypnotic-naïve (94%), and 361,012 antipsychotic or mania medicine-naïve (98%) patients. Overall, patient characteristics did not vary meaningfully by critical care status within the psychotropic-naïve, antidepressant-naïve, anxiolytic and hypnotic-naïve, and antipsychotic and mania medicine-naïve groups (Supplemental Tables S1–S3).

### Early (0–90 day) incident new psychotropic prescriptions after hospital discharge in psychotropic-naïve patients

**Figure 3.**
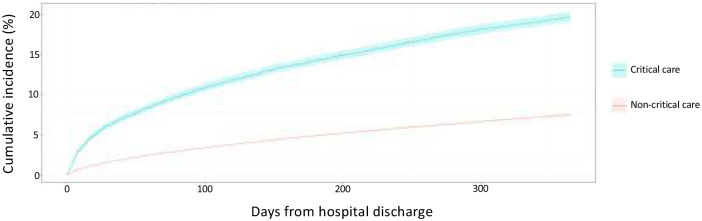
Unadjusted cumulative incidence for new psychotropic (any) prescription within 365 days of hospital discharge in psychotropic-naïve patients.^a^ ^a^Psychotropic-naïve patients are those that did not receive a community prescription for a psychotropic within 180 days prior to index hospitalisation.

Among psychotropic-naïve survivors, 10.3% (1610/15,609) of critical care and 3.2% (9743/307,429) of non-critical care survivors received at least one new psychotropic prescription within 90 days of hospital discharge. By 365 days, 19.7% of critical care and 7.5% of non-critical care survivors received at least one prescription for a new psychotropic (Figure 3). For each psychotropic category, there was a higher incidence of new prescription in the critical care group compared to the non-critical care group within 90 days (antidepressants: 6.1%vs 2.3%; anxiolytics or hypnotics: 6.5%vs 1.8%; and antipsychotics or mania medicine: 1.1%vs 0.5%) (Supplemental Figure S3). Z-drug hypnotics (e.g. zopiclone, zolpidem, and zaleplon) was the drug class prescribed most frequently to 3.5% of critical care survivors compared to 0.6% of non-critical care survivors (Table 2). Among those prescribed any psychotropic, prescriptions for more than one psychotropic group were more common in critical care survivors (critical care 14% (231/1610) vs non-critical care 12% (1208/9743)) (Supplemental Figure S2).

### Associations between critical care hospitalisation and early (0–90 day) new psychotropic prescriptions

In unadjusted analysis restricted to psychotropic-naïve patients, critical care was associated with a greater than three-fold increase in risk of any new psychotropic prescription within 90 days of hospital discharge (unadjusted HR 3.39; 95% CI: 3.22–3.57). After adjustment for confounders, this association was attenuated but remained significantly elevated (adjusted HR 2.03; 95% CI: 1.91–2.16). For each psychotropic class, naïve critical care survivors had a higher risk of prescription than naïve hospitalised non-critical care survivors (antidepressants adjusted HR 1.86 95% CI: 1.73–2.00; anxiolytics/hypnotics adjusted HR 2.18, 95% CI: 2.04–2.34; antipsychotics/mania medicines adjusted HR 1.22 95% CI: 1.06–1.41) (Table 3).

**Table 3.** Association between critical care hospitalisation and new psychotropic prescription within 90 days of hospital discharge in hospital survivors.

Psychotropic medication class	Number of new prescription/naïve critical care survivors^a^ (%; 95% CI)	Number of new prescription/naïve non-critical care survivors^a^ (%; 95% CI)	Unadjusted HR (95% CI)	Adjusted HR^b^ (95% CI)
New psychotropic medication (any)	1610/15,609 (10.3; 9.8–10.8)	9743/307,429 (3.2; 3.1–3.2)	3.39 (3.22–3.57)	2.03 (1.91–2.16)
New antidepressant	1048/17,207 (6.1; 5.7–6.5)	7232/317,708 (2.3; 2.2–2.3)	2.73 (2.56–2.91)	1.86 (1.73–2.00)
New anxiolytic or hypnotic	1307/20,108 (6.5; 6.2–6.8)	6194/346,609 (1.8; 1.7–1.8)	3.74 (3.52–3.97)	2.18 (2.04–2.34)
New antipsychotic or mania medication	247/22,412 (1.1; 1.0–1.2)	1787/361,012 (0.5; 0.5–0.5)	2.23 (1.95–2.55)	1.22 (1.06–1.41)

CI: confidence interval; HR: hazard ratio.

a Patients were only included in the denominator if they had not have received a community prescription for the specific medication category within 180 days prior to index hospitalisation.

b Adjusted for sex, age group, ethnicity, Scottish Index of Multiple Deprivation (SIMD) quintile, main condition at hospital admission, index morbidity category (5 years prior to hospitalisation), previous psychiatric admission category (5 years prior to hospitalisation), previous hospital admission category (1 year prior to hospitalisation), and previous emergency attendances category (1 year prior to hospitalisation).

### Late (91–365 day) incident new psychotropic prescriptions after hospital discharge

Among patients that were alive and psychotropic-naïve at 90 days after index hospital discharge, 10.8% of critical care (1467/13,594) and 4.6% of non-critical care survivors (13,312/288,222) were prescribed a new psychotropic between 91 and 365 days post-discharge. Unadjusted HR for the association of critical care and new prescription was 2.42 (95% CI: 2.29–2.55). After accounting for confounders, the adjusted HR was 1.38 (95% CI: 1.30–1.46). Findings were similar across psychotropic groups (Supplemental Table S5).

### Effect modification analyses for early (0–90 day) new psychotropic prescriptions

Significant interaction terms were observed for sex, SIMD, and comorbidity category (Supplemental Table S6). In patients without comorbidities on index hospitalisation, critical care survivors had a greater risk of 90 days incident psychotropic prescription compared to non-critical care survivors (adjusted HR 3.75, 95% CI: 3.47–4.05), in contrast to those with two or more comorbidities (adjusted HR 1.24, 1.12–1.36). There was also a higher magnitude of association in males (vs females). Variation in HRs across SIMD quintiles was non-monotonic.

### Sensitivity analyses

Analyses excluding patients who died in the first 30 days from hospital discharge were consistent with the primary analysis (any new psychotropic prescription within 90 days in critical care vs non-critical care, adjusted HR 2.09; 95% CI: 1.97–2.22). There was a slight attenuation towards the null in analyses excluding antidepressants and/or antidepressant daily doses commonly used for chronic pain (adjusted HR 1.69; 95% CI: 1.54–1.85). Lastly, when patients were censored on hospital readmission after index discharge, the adjusted HRs increased slightly across all psychotropic groups (Supplemental Table S7).

## Discussion

Our study demonstrates that one in three critical care survivors received a psychotropic prescription within 90 days of hospital discharge, compared to one in seven non-critical care survivors. This increased risk for critical care survivors was evident across all three psychotropic classes and in patients previously naïve to psychotropic medicines. Of note, one in seven critical care survivors received a prescription for benzodiazepines or other hypnotics/anxiolytics within 90 days of hospital discharge. After adjusting for confounders, the risk of any new psychotropic prescription was double that in critical care survivors compared to non-critical care survivors. Interestingly, this risk was highest among patients without comorbidities at index hospitalisation. In the later follow-up period (91–365 days), the risk of new psychotropic prescription remained significantly elevated in critical care survivors compared to non-critical care hospitalised survivors, albeit lower in magnitude.

We compare our study to similar studies based on data from Denmark, Canada, and the Netherlands. However, it is important to recognise that prescribing practices frequently differ as demonstrated by a 2016 study^12^ which highlighted the heterogeneity in prescribing psychotropics across seven European countries (not restricted to hospitalised patients). Our findings are comparable to a study by Wunsch et al. that found an incidence of new psychotropic prescription within 90 days of hospital discharge in 12.7% (1261/9912) of critical care survivors (limited to mechanically ventilated patients) versus 5.0% of the hospitalised comparator group in Denmark between 2006 and 2008 (adjusted HR 2.5).^13^ Like our study, they also found hypnotics to be prescribed most frequently in the first 90 days after hospital discharge. Their study excluded more patients by excluding those with previous psychotropic prescriptions within 5 years of hospitalisation, compared to 180 days in our study. Next, a large population-based study on data from Manitoba, Canada found the prevalence of all psychotropic medicines in the year after critical care discharge to be higher than the year prior to admission,^14^ where we found the overall prevalence to be similar before and after hospitalisation. A recent study from Ontario, Canada of sedative-naïve critical care survivors aged 66 years and over demonstrated that 3.5% were prescribed benzodiazepines, 1.1% non-benzodiazepine sedatives, and 1.1% antipsychotics within just 7 days of hospital discharge, comparable to our study figures at 90 days after hospital discharge.^15^ Lastly, a survey sent to 1-year survivors who had a critical care stay in one hospital in the Netherlands found that 15% of respondents reported psychotropic medicine use within 1 year after hospital discharge.^16^ Focusing on antipsychotics, a single-centre prospective study of ICU patients in the US treated for acute respiratory failure or shock found that 10% (42/412) of antipsychotic-naïve survivors were discharged from the hospital on antipsychotics - all after initiation in the ICU.^17^ This is substantially higher than our finding of 1.1% of antipsychotic-naïve critical care survivors prescribed antipsychotics or mania medicines after hospital discharge and the recent published findings from Canada.^15^ The restriction of their study to patients with respiratory failure and/or shock may explain at least part of the discordance, as delirium and subsequent antipsychotic treatment would likely be higher in those patients. Nonetheless, inappropriate antipsychotic continuation after critical care in the Lothian region is unlikely to occur based on our results.

We found that patients without index comorbidity had a higher risk of new psychotropic prescription associated with critical care admission than those with one or more index comorbidities. Previous research on adult survivors of critical illness in Scotland has demonstrated similar findings in a range of broader post-index discharge outcomes.^18^

Over half of critical illness survivors manifest some form of psychiatric morbidity,^19^ and symptoms of mental health disorders are associated with worse outcomes, functional impairment, increased healthcare utilisation, and decreased quality of life in medically-unwell populations.^20^ Our study has shown that anxiolytics and hypnotics (largely made up of benzodiazepines and z-drugs commonly used to treat anxiety and sleep disorders) are regularly prescribed to critical care survivors in Scotland. This is concerning because of the increased risk of falls, fractures, and physical dependence are serious adverse events associated with benzodiazepines^21^ and z-drugs, especially in older adults.^22–24^ Survivors of critical illness may have newly acquired impairments in physiological and functional reserve, potentially increasing the risk of adverse events further.^25,26^ Moreover, benzodiazepines are implicated in most drug-related deaths reported in Scotland.^27^ Further investigation is needed to understand if the prescribing of psychotropic medicines is due to unintended continuation after critical care or as pharmacologic treatment of new mental health or sleep issues after critical illness. Future research should focus on the safety of psychotropics in survivors of critical illness to help guide policy for clinical practice, including how increased psychotropic medicine exposure may contribute to the high unplanned hospital readmission risk of critical care survivors.^28^

Our study has several strengths. The data sources are population-based over 8 years and therefore have low risk for selection bias. We included all hospitalised adults without restriction lending to greater generalisability amongst hospitalised patients. Our data sources allowed comparison of psychotropic prescribing after critical illness to a non-critical care hospitalised comparator group so that we were able to estimate the risk of new prescriptions associated with critical care. This study also has some limitations. Our data source did not allow us to distinguish medicines started during hospitalisation and continued after discharge from those prescribed after hospitalisation without initiation in hospital. While we had community prescriptions (more than 95% of which are prescribed by GPs^29^), we did not have other GP data (e.g. diagnosis codes). However, past studies report severe underestimates of mental health disorders based on diagnosis codes when compared to psychotropic prescriptions.^16,30,31^ As our study was centred on a region of Scotland, prescribing information on patients who moved into Lothian shortly before hospitalisation or left Lothian after hospitalisation could be misclassified; however, this is likely a small portion of the study population (e.g. in 2019, 87,400 people (1.6%) moved to Scotland and 57,100 people (1.0%) emigrated).^32^ Additionally, our study population only included patients who presented to the ED at least once during the study period – patients who were admitted to the critical care units via other means (e.g. complications after planned hospital admission), were not included if they did not also attend a Lothian ED at least once during the study period, potentially leading to selection bias. Finally, our definition of psychotropic medicines excluded gabapentinoids, increasingly used as analgesia adjuncts,^33–36^ as well as part of pharmacologic treatment for anxiety^37,38^ and insomnia.^38^

## Conclusions

Of all critical care survivors, one-third received a psychotropic prescription within 90 days after hospital discharge while one in 10 psychotropic-naïve critical care survivors received a new psychotropic prescription. Benzodiazepines and other hypnotic medicines were commonly prescribed to critical care survivors. The risk of incident psychotropic prescription within 90 days of hospital discharge was two-fold higher among critical care survivors compared to non-critical care survivors, and more pronounced in those without index comorbidities. Future research should focus on the requirement for and safety of psychotropic medicines in survivors of critical illness, to help guide policy for clinical practice.

## Supplemental Material

sj-docx-1-inc-10.1177_17511437231223470 – Supplemental material for Psychotropic prescribing after hospital discharge in survivors of critical illness, a retrospective cohort study (2012–2019) Supplemental material, sj-docx-1-inc-10.1177_17511437231223470 for Psychotropic prescribing after hospital discharge in survivors of critical illness, a retrospective cohort study (2012–2019) by Elizabeth T Mansi, Christopher T Rentsch, Richard S Bourne, Bruce Guthrie and Nazir I Lone in Journal of the Intensive Care Society
