# Supplementary material for: Psychotropic prescribing after hospital discharge in survivors of critical illness, a retrospective cohort study (2012–2019)
Source: J Intensive Care Soc. 2024 Jan 19;25(2):171–80. doi: 10.1177/17511437231223470 (PMC11081855; doi:10.1177/17511437231223470)
Supplement: sj-docx-1-inc-10.1177_17511437231223470 – Supplemental material for Psychotropic prescribing after hospital discharge in survivors of critical illness, a retrospective cohort study (2012–2019) [file sj-docx-1-inc-10.1177_17511437231223470.docx]

**Psychotropic prescribing after hospital discharge in survivors of critical illness, a retrospective cohort study (2012-2019)**

Elizabeth T. Mansi DVM MPH^1^, Christopher T. Rentsch PhD MPH^2,3^, Richard S. Bourne MSc MRes PhD^4,5^, Bruce Guthrie MB BChir PhD^1,6^, Nazir I. Lone MBChB MSc PhD FFICM^1,7^

^1^ Usher Institute, University of Edinburgh, Edinburgh, UK

^2^ Faculty of Epidemiology and Population Health, London School of Hygiene & Tropical Medicine, London, UK

^3^ Department of Internal Medicine, Yale School of Medicine, New Haven, USA

^4^ Departments of Pharmacy and Critical Care, Sheffield Teaching Hospitals NHS Foundation Trust, Sheffield, UK

^5^ Division of Pharmacy and Optometry, School of Health Sciences, Faculty of Biology, Medicine and Health, The University of Manchester, Manchester, UK

^6^ Advanced Care Research Centre, University of Edinburgh, Edinburgh, UK

^7^ University Department of Anaesthesia, Critical Care, and Pain Medicine, School of Clinical Sciences, University of Edinburgh, Edinburgh, UK

Supplementary Material

[Appendix A. British National Formulary (BNF) select subsections, generic names, and BNF codes. 2](#_Toc145672115)

[Supplementary Table S1. Characteristics of the critical care and non-critical care hospitalised antidepressant-naïve^a^ survivors in Lothian 2012-2019. 5](#_Toc145672116)

[Supplementary Table S2. Characteristics of the critical care and non-critical care hospitalised anxiolytic and hypnotic-naïve^a^ survivors in Lothian 2012-2019. 6](#_Toc145672117)

[Supplementary Table S3. Characteristics of the critical care and non-critical care hospitalised antipsychotic and mania medicine-naïve survivors^a^ in Lothian 2012-2019. 7](#_Toc145672118)

[Supplementary Table S4. Multivariable model of the association between critical care hospitalisation and new psychotropic prescription within 90 days of hospital discharge. 8](#_Toc145672119)

[Supplementary Table S5. Association between critical care hospitalisation and new psychotropic prescription within 365 days of hospital discharge in hospital survivors *(amongst those that survived to 91 days and did not receive a prescription within the first 90 days after hospital discharge*). 9](#_Toc145672120)

[Supplementary Table S6. Effect modification analyses for the association between critical care hospitalisation and new psychotropic prescription within 90 days of hospital discharge in hospital survivors. 10](#_Toc145672121)

[Supplementary Table S7. Sensitivity analyses for the association between critical care hospitalisation and new psychotropic prescription within 90 days of hospital discharge, *censoring for hospital readmission*. 11](#_Toc145672122)

[Supplementary Figure S1. Hospitalised survivors prescribed psychotropic medications within 90 days after critical care (n=7527, left) and non-critical care hospitalisation (n=54,589, right). 12](#_Toc145672123)

[Supplementary Figure S2. Psychotropic-naïve^a^ hospitalised survivors prescribed psychotropic medications within 90 days after critical care (n=1610, left) and non-critical care hospitalisation (n=9743, right). 12](#_Toc145672124)

[Supplementary Figure S3. Cumulative incidence of new (A) antidepressant; (B) anxiolytic or hypnotic; and (C) antipsychotic or mania medication prescription within 90 days of hospital discharge in critical care vs non-critical care medication-naïve^a^ patients. 13](#_Toc145672125)

### Appendix A. British National Formulary (BNF) select subsections, generic names, and BNF codes.

**Subsection 4.1: Hypnotics and anxiolytics**

**4.1.1: Hypnotics**

1. Chloral Hydrate (0401010B0)
2. Clomethiazole (0401010F0)
3. Clomethiazole Edisilate (0401010D0)
4. Cloral Betaine (0401010C0)
5. Flurazepam Hydrochloride (0401010L0)
6. Loprazolam Mesilate (0401010N0)
7. Lormetazepam (0401010P0)
8. Melatonin (0401010AD)
9. Midazolam Maleate (0401010Q0)
10. Nitrazepam (0401010R0)
11. Other Hypnotic Preps (040101000)
12. Sodium Oxybate (0401010AC)
13. Temazepam (0401010T0)
14. Triclofos Sodium (0401010X0)
15. Zaleplon (0401010W0)
16. Zolpidem Tartrate (0401010Y0)
17. Zopiclone (0401010Z0)

**4.1.2: Anxiolytics**

1. Alprazolam (0401020A0)
2. Bromazepam (0401020G0)
3. Buspirone Hydrochloride (0401020B0)
4. Chlordiazepoxide Hydrochloride (0401020E0)
5. Diazepam (0401020K0)
6. Lorazepam (0401020P0)
7. Meprobamate (0401020R0)
8. Oxazepam (0401020T0)

**Subsection 4.2: Drugs used in psychoses and related disorders**

**4.2.1: Antipsychotic drugs**

1. Amisulpride (0402010A0)
2. Aripiprazole (0402010AD)
3. Benperidol (0402010B0)
4. Cariprazine (0402010AJ)
5. Chlorpromazine Hydrochloride (0402010D0)
6. Chlorprothixene (0402010F0)
7. Clozapine (0402010C0)
8. Flupentixol Hydrochloride (0402010H0)*
9. Fluphenazine Hydrochloride (0402010I0)
10. Haloperidol (0402010J0)
11. Levomepromazine hydrochloride (0402010L0) – *excluded from analysis*
12. Levomepromazine maleate (0402010K0) – *excluded from analysis*
13. Loxapine Succinate (0402010M0)
14. Lurasidone (0402010AI)
15. Melperone Hydrochloride (0402010AF)
16. Olanzapine (040201060)
17. Paliperidone (0402010AE)
18. Pericyazine (0402010P0)
19. Perphenazine (0402010Q0)
20. Pimozide (0402010R0)
21. Promazine Hydrochloride (0402010S0)
22. Quetiapine (0402010AB)
23. Risperidone (040201030)
24. Sulpiride (0402010U0)
25. Thioridazine (0402010W0)
26. Trifluoperazine (0402010X0)
27. Ziprasidone Hydrochloride (0402010AG)
28. Zotepine (0402010AC)
29. Zuclopenthixol Acetate (040201010)
30. Zuclopenthixol Hydrochloride (0402010T0)

**4.2.3: Drugs used for mania and hypomania**

1. Asenapine (0402030R0)
2. Lithium Carbonate (0402030K0)
3. Lithium Citrate (0402030P0)
4. Valproic Acid (0402030Q0)

**Subsection 4.3: Antidepressant drugs**

**4.3.1: Tricyclic and related antidepressants**

1. Amitriptyline hydrochloride (0403010B0)
2. Amoxapine (0403010C0)
3. Clomipramine hydrochloride (0403010F0)
4. Dosulepin hydrochloride (0403010J0)
5. Doxepin (0403010L0)
6. Imipramine hydrochloride (0403010N0)
7. Lofepramine hydrochloride (0403010R0)
8. Maprotiline hydrochloride (0403010S0)
9. Mianserin hydrochloride (0403010T0)
10. Nortriptyline (0403010V0)
11. Trazodone hydrochloride (0403010X0)
12. Trimipramine maleate (0403010Y0)

**4.3.2: Monoamine-oxidase inhibitors**

1. Isocarboxazid (0403020H0)
2. Moclobemide (0403020K0)
3. Phenelzine sulfate (0403020M0)
4. Tranylcypromine sulfate (0403020Q0)

**4.3.3: Selective serotonin reuptake inhibitors**

1. Citalopram hydrobromide (0403030D0)
2. Citalopram hydrochloride (0403030Z0)
3. Escitalopram (0403030X0)
4. Fluoxetine hydrochloride (0403030E0)
5. Fluvoxamine maleate (0403030L0)
6. Paroxetine hydrochloride (0403030P0)
7. Sertraline hydrochloride (0403030Q0)

**4.3.4: Other antidepressant drugs**

1. Agomelatine (0403040Z0)
2. Duloxetine hydrochloride (0403040Y0)
3. Flupentixol hydrochloride (0403040F0)*
4. Mirtazapine (0403040X0)
5. Nefazodone hydrochloride (0403040T0)
6. Oxitriptan (0403040R0)
7. Reboxetine (0403040U0)
8. Tryptophan (0403040S0)
9. Venlafaxine (0403040W0)
10. Vortioxetine (0403040AB)

* Flupentixol is the only one of the psychotropic medications listed in the BNF under more than one drug class (antidepressant and antipsychotic). As it is technically a first-generation antipsychotic, we categorised it only as such for our study purposes.

### Supplementary Table S1. Characteristics of the critical care and non-critical care hospitalised antidepressant-naïve^a^ survivors in Lothian 2012-2019.

| ***Characteristics*** | | **Critical care**  **n = 17,207 (%)** | **Non-critical care**  **n = 317,708 (%)** |
| --- | --- | --- | --- |
| **Sex** | **Female**  **Male** | 6670 (38.8)  10,537 (61.2) | 160,046 (50.4)  157,662 (49.6) |
| **Age** | **Median (IQR)** | 63 (48 to 74) | 57 (37 to 74) |
| **Age Group** | **18-29**  **30-39**  **40-49**  **50-59**  **60-69**  **70-79**  **80+** | 1428 (8.3)  1355 (7.9)  1775 (10.3)  2722 (15.8)  3787 (22.0)  3942 (22.9)  2198 (12.8) | 49,069 (15.4)  37,559 (11.8)  39,769 (12.5)  45,572 (14.3)  45,573 (14.3)  47,818 (15.1)  52,348 (16.5) |
| **Ethnicity** | **Asian**  **Black**  **Mixed or Other**  **White**  **Missing** | 230 (1.6)  97 (0.7)  70 (0.5)  14,282 (97.3)  2528 | 5322 (2.0)  1707 (0.6)  1850 (0.7)  262,657 (96.7)  46,172 |
| **Socioeconomic Deprivation** | **1 (most deprived)**  **2**  **3**  **4**  **5 (least deprived)**  **Missing** | 2516 (14.8)  4057 (23.8)  3117 (18.3)  2992 (17.6)  4341 (25.5)  184 | 46,223 (14.7)  75,379 (24.0)  56,907 (18.1)  56,373 (18.0)  78,847 (25.1)  3979 |
| **Year of Hospital Discharge** | **2012**  **2013**  **2014**  **2015**  **2016**  **2017**  **2018**  **2019**  **2020** | 2012 (11.7)  2172 (12.6)  2324 (13.5)  2172 (12.6)  2118 (12.3)  2136 (12.4)  2132 (12.4)  2052 (11.9)  89 (0.5) | 39,561 (12.5)  38,949 (12.3)  40,704 (12.8)  39,381 (12.4)  36,821 (11.6)  37,822 (11.9)  38,603 (12.2)  45,194 (14.2)  673 (0.2) |
| **Main Condition at Hospitalisation** | **Circulatory**  **Neoplasms**  **Injury or Poisoning**  **Digestive**  **Respiratory**  **Abnormal Findings^b^**  **Genitourinary**  **Musculoskeletal**  **Infectious**  **All Other Conditions** | 3846 (22.4)  3553 (20.6)  2454 (14.3)  2529 (14.7)  1564 (9.1)  476 (2.8)  708 (4.1)  293 (1.7)  451 (2.6)  1333 (7.7) | 31,891 (10.0)  17,922 (5.6)  54,714 (17.2)  31,581 (9.9)  25,193 (7.9)  44,453 (14.0)  21,882 (6.9)  22,304 (7.0)  7553 (2.4)  60,215 (19.0) |
| **Length of Hospital Stay (days)** | **Mean (SD)**  **Median (IQR)** | 19.9 (39.1)  9 (5 to 18) | 5.4 (21.1)  1 (0 to 4) |
| **Number Comorbidities at Hospital Admit** | **0**  **1**  **2+** | 8653 (50.3)  2987 (17.4)  5567 (32.4) | 275,011 (86.6)  21,047 (6.6)  21,650 (6.8) |
| **Previous Psychiatric Admission** |  | 295 (1.7) | 1908 (0.6) |
| **Number Hospital Admissions** | **0**  **1**  **2+** | 8822 (51.3)  4136 (24.0)  4249 (24.7) | 279,297 (87.9)  24,768 (7.8)  13,643 (4.3) |
| **Number ED Attendances** | **0**  **1**  **2+** | 10,525 (61.2)  4356 (25.3)  2326 (13.5) | 256,887 (80.9)  44,145 (13.9)  16,676 (5.2) |
| **Died within 90 days of Hospital Discharge** |  | 557 (3.2) | 10,771 (3.4) |

*^a^* Antidepressant-naïve patients are those that did not receive a community prescription for an antidepressant within 180 days prior to index hospitalisation. ^b^ Abnormal findings: International Classification of Diseases-10 codes R00-R99 (“Symptoms, signs and abnormal clinical and laboratory findings, not elsewhere classified”).

*SD*: standard deviation; *IQR*: interquartile range; *ED*: emergency department

### Supplementary Table S2. Characteristics of the critical care and non-critical care hospitalised anxiolytic and hypnotic-naïve^a^ survivors in Lothian 2012-2019.

| ***Characteristics*** | | **Critical care**  **n = 20,108 (%)** | **Non-critical care**  **n = 346,609 (%)** |
| --- | --- | --- | --- |
| **Sex** | **Female**  **Male** | 8437 (42.0)  11,671 (58.0) | 180,150 (52.0)  166,459 (48.0) |
| **Age** | **Median (IQR)** | 63 (49 to 73) | 56 (38 to 74) |
| **Age Group** | **18-29**  **30-39**  **40-49**  **50-59**  **60-69**  **70-79**  **80+** | 1601 (8.0)  1533 (7.6)  2152 (10.7)  3356 (16.7)  4553 (22.6)  4499 (22.4)  2414 (12.0) | 52,568 (15.2)  40,632 (11.7)  44,187 (12.7)  50,845 (14.7)  49,982 (14.4)  51,822 (16.3)  56,573 (16.3) |
| **Ethnicity** | **Asian**  **Black**  **Mixed or Other**  **White**  **Missing** | 251 (1.5)  110 (0.6)  79 (0.5)  16,822 (97.5)  2846 | 5562 (1.9)  1794 (0.6)  1956 (0.7)  287,355 (96.9)  49,942 |
| **Socioeconomic Deprivation** | **1 (most deprived)**  **2**  **3**  **4**  **5 (least deprived)**  **Missing** | 3096 (15.6)  4975 (25.0)  3637 (18.3)  3394 (17.1)  4798 (24.1)  208 | 51,289 (15.0)  83,572 (24.4) 61,992 (18.1)  61,199 (17.9)  84,344 (24.6)  4213 |
| **Year of Hospital Discharge** | **2012**  **2013**  **2014**  **2015**  **2016**  **2017**  **2018**  **2019**  **2020** | 2297 (11.4)  2484 (12.4)  2671 (13.3)  2547 (12.7)  2485 (12.4)  2506 (12.5)  2580 (12.8)  2433 (12.1)  105 (0.5) | 42,649 (12.3)  42,104 (12.1)  43,874 (12.7)  42,975 (12.4)  40,242 (11.6)  41,431 (12.0)  42,479 (12.3)  50,111 (14.5)  744 (0.2) |
| **Main Condition at Hospitalisation** | **Circulatory**  **Neoplasms**  **Injury or Poisoning**  **Digestive**  **Respiratory**  **Abnormal Findings^b^**  **Genitourinary**  **Musculoskeletal**  **Infectious**  **All Other Conditions** | 4317 (21.5)  3904 (19.4)  3054 (15.2)  3058 (15.2)  1834 (9.1)  603 (3.0)  840 (4.2)  398 (2.0)  535 (2.7)  1565 (7.8) | 33,833 (9.8)  18,920 (5.5)  59,559 (17.2)  34,317 (9.9)  27,408 (7.9)  48,477 (14.0)  24,384 (7.0)  25,176 (7.3)  8211 (2.4)  66,324 (19.1) |
| **Length of Hospital Stay (days)** | **Mean (SD)**  **Median (IQR)** | 20.1 (38.8)  9 (5 to 19) | 5.4 (21.0)  1 (0 to 3) |
| **Number Comorbidities at Hospital Admit** | **0**  **1**  **2+** | 9928 (49.4)  3547 (17.6)  6633 (33.0) | 294,103 (84.9)  25,534 (7.4)  26,972 (7.8) |
| **Previous Psychiatric Admission** |  | 463 (2.3) | 2759 (0.8) |
| **Number Hospital Admissions** | **0**  **1**  **2+** | 10,320 (51.3)  4819 (24.0)  4969 (24.7) | 299,895 (86.5)  30,228 (8.7)  16,486 (4.8) |
| **Number ED Attendances** | **0**  **1**  **2+** | 12,071 (60.0)  5142 (25.6)  2895 (14.4) | 274,292 (79.1)  51,641 (14.9)  20,676 (6.0) |
| **Died within 90 days of Hospital Discharge** |  | 628 (3.1) | 11,521 (3.3) |

*^a^* Anxiolytic and hypnotic-naïve patients are those that did not receive a community prescription for an anxiolytic or hypnotic within 180 days prior to index hospitalisation. ^b^*Abnormal findings:* International Classification of Diseases-10 codes R00-R99 (“Symptoms, signs and abnormal clinical and laboratory findings, not elsewhere classified”).

*SD*: standard deviation; *IQR*: interquartile range; *ED*: emergency department.

### Supplementary Table S3. Characteristics of the critical care and non-critical care hospitalised antipsychotic and mania medicine-naïve survivors^a^ in Lothian 2012-2019.

| ***Characteristics*** | | **Critical care**  **n = 22,412 (%)** | **Non-critical care**  **n = 361,012 (%)** |
| --- | --- | --- | --- |
| **Sex** | **Female**  **Male** | 9752 (43.5)  12,660 (56.5) | 189,543 (52.5)  171,469 (47.5) |
| **Age** | **Median (IQR)** | 63 (49 to 73) | 56 (38 to 74) |
| **Age Group** | **18-29**  **30-39**  **40-49**  **50-59**  **60-69**  **70-79**  **80+** | 1674 (7.5)  1741 (7.8)  2500 (11.2)  3821 (17.0)  5030 (22.4)  4986 (22.2)  2660 (11.9) | 53,753 (14.9)  42,553 (11.8)  46,695 (12.9)  53,293 (14.8)  52,130 (14.4)  53,952 (14.9)  58,636 (16.2) |
| **Ethnicity** | **Asian**  **Black**  **Mixed or Other**  **White**  **Missing** | 269 (1.4)  112 (0.6)  86 (0.4)  18,854 (97.6)  3091 | 5698 (1.8)  1842 (0.6)  2012 (0.7)  299,782 (96.9)  51,678 |
| **Socioeconomic Deprivation** | **1 (most deprived)**  **2**  **3**  **4**  **5 (least deprived)**  **Missing** | 3491 (15.7)  5603 (25.3)  4069 (18.3)  3758 (16.9)  5267 (23.7)  224 | 53,755 (15.1)  87,346 (24.5)  64,719 (18.1)  63,374 (17.8)  87,494 (24.5)  4324 |
| **Year of Hospital Discharge** | **2012**  **2013**  **2014**  **2015**  **2016**  **2017**  **2018**  **2019**  **2020** | 2587 (11.5)  2797 (12.5)  2967 (13.2)  2852 (12.7)  2776 (12.4)  2794 (12.5)  2848 (12.7)  2677 (11.9)  114 (0.5) | 44,725 (12.4)  43,928 (12.2)  45,727 (12.70  44,845 (12.4)  41,963 (11.6)  43,079 (11.9)  44,022 (12.2)  51,969 (14.4)  754 (0.2) |
| **Main Condition at Hospitalisation** | **Circulatory**  **Neoplasms**  **Injury or Poisoning**  **Digestive**  **Respiratory**  **Abnormal Findings^b^**  **Genitourinary**  **Musculoskeletal**  **Infectious**  **All Other Conditions** | 4649 (20.7)  4478 (20.0)  3354 (15.0)  3367 (15.0)  2136 (9.5)  673 (3.0)  950 (4.2)  470 (2.1)  610 (2.7)  1725 (7.7) | 34,868 (9.7)  20,255 (5.6)  61,332 (17.0)  35,642 (9.9)  28,667 (7.9)  50,422 (14.0)  25,354 (7.0)  26,717 (7.4)  8503 (2.4)  69,252 (19.2) |
| **Length of Hospital Stay (days)** | **Mean (SD)**  **Median (IQR)** | 20.1 (37.4)  9 (5 to 19) | 5.4 (37.4)  1 (0 to 3) |
| **Number Comorbidities at Hospital Admit** | **0**  **1**  **2+** | 10,669 (47.6)  3993 (17.8)  7750 (34.6) | 302,135 (83.7)  28,051 (7.8)  30,826 (8.5) |
| **Previous Psychiatric Admission** |  | 472 (2.1) | 2637 (0.7) |
| **Number Hospital Admissions** | **0**  **1**  **2+** | 11,100 (49.5)  5389 (24.0)  5923 (26.4) | 308,760 (85.5)  32,988 (9.1)  19,264 (5.3) |
| **Number ED Attendances** | **0**  **1**  **2+** | 13,195 (58.9)  5763 (25.7)  3454 (15.4) | 282,086 (78.1)  55,762 (15.4)  23,164 (6.4) |
| **Died within 90 days of Hospital Discharge** |  | 733 (3.3) | 12,283 (3.4) |

*^a^* Antipsychotic and mania medicine-naïve patients are those that did not receive a community prescription for an antipsychotic or mania medicine within 180 days prior to index hospitalisation. ^b^ *Abnormal findings:* International Classification of Diseases-10 codes R00-R99 (“Symptoms, signs and abnormal clinical and laboratory findings, not elsewhere classified”).

*SD*: standard deviation; *IQR*: interquartile range; *ED*: emergency department

### Supplementary Table S4. Multivariable model of the association between critical care hospitalisation and new psychotropic prescription within 90 days of hospital discharge.

| ***Characteristics*** | | **Any new psychotropic prescription in psychotropic-naïve patients^a^** | **Any new antidepressant prescription in antidepressant-naïve patients^a^** | **Any new anxiolytic or hypnotic prescription in anxiolytic and hypnotic-naïve patients^a^** | **Any new antipsychotic or mania prescription in antipsychotic and mania medication-naïve patients^a^** |
| --- | --- | --- | --- | --- | --- |
|  |  | Adjusted HR^b^  (95% CI) | Adjusted HR  (95% CI) | Adjusted HR  (95% CI) | Adjusted HR  (95% CI) |
| **Hospitalisation** | **Critical care**  **Non-critical care** | 2.03 (1.91-2.16)  Reference | 1.86 (1.73-2.00)  Reference | 2.18 (2.04-2.34)  Reference | 1.22 (1.06-1.41)  Reference |
| **Sex** | **Female**  **Male** | 1.30 (1.25-1.35)  Reference | 1.48 (1.41-1.54)  Reference | 1.30 (1.24-1.36)  Reference | - 1. (0.92-1.10)   Reference |
| **Age Group** | **18-29**  **30-39**  **40-49**  **50-59**  **60-69**  **70-79**  **80+** | Reference  1.06 (0.99-1.14)  1.03 (0.96-1.11)  1.01 (0.94-1.08)  0.84 (0.78-0.90)  0.83 (0.77-0.89)  0.91 (0.85-0.98) | Reference  1.02 (0.94-1.11)  0.99 (0.91-1.07)  0.94 (0.87-1.01)  0.72 (0.66-0.78)  0.68 (0.62-0.73)  0.74 (0.68-0.80) | Reference  1.29 (1.17-1.42)  1.34 (1.22-1.46)  1.19 (1.09-1.30)  1.03 (0.94-1.13)  1.03 (0.94-1.13)  0.95 (0.87-1.04) | 0.90 (0.74-1.08)  0.85 (0.71-1.02)  0.73 (0.61-0.88)  0.74 (0.61-0.89)  1.14 (0.96-1.35)  2.06 (1.77-2.41) |
| **Ethnicity** | **Asian**  **Black**  **Mixed or Other**  **White** | 0.84 (0.72-0.98)  0.71 (0.53-0.94)  1.02 (0.96-1.07)  Reference | 0.87 (0.73-1.03)  0.66 (0.47-0.93)  1.04 (0.97-1.11)  Reference | 0.67 (0.54-0.84)  0.56 (0.37-0.85)  1.02 (0.95-1.09)  Reference | 0.78 (0.49-1.24)  1.28 (0.69-2.40)  1.19 (1.05-1.35)  Reference |
| **Socioeconomic Deprivation** | **1 (most deprived)**  **2**  **3**  **4**  **5 (least deprived)** | 1.16 (1.10-1.24)  1.12 (1.06-1.18)  1.09 (1.03-1.15)  0.97 (0.92-1.03)  Reference | 1.24 (1.16-1.33)  1.19 (1.11-1.26)  1.11 (1.04-1.19)  1.00 (0.93-1.07)  Reference | 1.15 (1.07-1.24)  1.10 (1.03-1.17)  1.13 (1.05-1.21)  1.02 (0.95-1.10)  Reference | 1.08 (0.93-1.24)  0.98 (0.87-1.12)  0.99 (0.87-1.14)  0.93 (0.81-1.07)  Reference |
| **Main Condition at Hospitalisation** | **Circulatory**  **Neoplasms**  **Injury or Poisoning**  **Digestive**  **Respiratory**  **Abnormal Findings^c^**  **Genitourinary**  **Musculoskeletal**  **Infectious**  **All Other Conditions** | Reference  1.12 (1.03-1.21  1.01 (0.93-1.08)  0.79 (0.73-0.86)  0.79 (0.72-0.87)  1.11 (1.03-1.20)  0.84 (0.76-0.92)  1.25 (1.14-1.36)  0.85 (0.74-0.98)  0.97 (0.90-1.04) | Reference  0.81 (0.73-0.91)  1.13 (1.03-1.23)  0.83 (0.75-0.92)  0.71 (0.64-0.80)  1.24 (1.14-1.36)  0.92 (0.82-1.03)  1.31 (1.18-1.46)  0.91 (0.77-1.07)  1.04 (0.95-1.13) | Reference  1.34 (1.22-1.47)  0.94 (0.86-1.03)  0.76 (0.68-0.84)  0.88 (0.79-0.98)  0.97 (0.88-1.07)  0.72 (0.64-0.81)  1.25 (1.12-1.39)  0.80 (0.67-0.96)  0.87 (0.79-0.95) | Reference  2.93 (2.35-3.65)  2.45 (2.00-3.00)  1.06 (0.82-1.37)  1.67 (1.32-2.11)  1.81 (1.46-2.25)  1.49 (1.15-1.94)  0.66 (0.46-0.93)  1.82 (1.30-2.55)  1.92 (1.56-2.36) |
| **Number Comorbidities at Hospital Admit** | **0**  **1**  **2+** | Reference  2.08 (1.96-2.20)  2.23 (2.10-2.38) | Reference  2.03 (1.90-2.17)  1.99 (1.84-2.14) | Reference  2.03 (1.89-2.18)  2.14 (1.99-2.30) | 2.11 (1.85-2.42)  2.50 (2.19-2.86) |
| **Previous Psychiatric Admission** | **None**  **Yes** | Reference  2.49 (2.18-2.84) | Reference  1.87 (1.62-2.15) | Reference  2.11 (1.86-2.40) | Reference  8.44 (7.27-9.79) |
| **Number Hospital Admissions** | **0**  **1**  **2+** | Reference  1.32 (1.25-1.40)  1.21 (1.12-1.30) | Reference  1.37 (1.28-1.47)  1.24 (1.14-1.35) | Reference  1.24 (1.16-1.33)  1.24 (1.14-1.35) | Reference  1.04 (0.91-1.19)  1.07 (0.92-1.25) |
| **Number ED Attendances** | **0**  **1**  **2+** | Reference  1.96 (1.87-2.06)  2.27 (2.13-2.41) | Reference  1.96 (1.86-2.07)  2.28 (2.12-2.45) | Reference  1.68 (1.59-1.78)  1.98 (1.83-2.13) | Reference  2.08 (1.86-2.32  2.46 (2.15-2.83 |

^a^ Patients were only included in the denominator if they had not have received a community prescription for the specific medication category within 180 days prior to index hospitalisation.

^b^ Adjusted for sex, age group, ethnicity, Scottish Index of Multiple Deprivation (SIMD) quintile, main condition at hospital admission, index morbidity category (five years prior to hospitalisation), previous psychiatric admission category (five years prior to hospitalisation), previous hospital admission category (one year prior to hospitalisation), and previous emergency attendances category (one year prior to hospitalisation). *CI:* confidence interval; *HR:* hazard ratio

^c^ *Abnormal findings:* International Classification of Diseases-10 codes R00-R99 (“Symptoms, signs and abnormal clinical and laboratory findings, not elsewhere classified”).

*HR:* hazard ratio; *CI:* confidence interval; *ED*: emergency department

### Supplementary Table S5. Association between critical care hospitalisation and new psychotropic prescription within 365 days of hospital discharge in hospital survivors *(amongst those that survived to 91 days and did not receive a prescription within the first 90 days after hospital discharge*).

| **Psychotropic medication class** | **Number of new prescription/naïve critical care survivors^a^**  **(%)** | **Number of new prescription/naïve non-critical care survivors^a^**  **(%)** | **Unadjusted HR (95% CI)** | **Adjusted HR (95% CI)^b^** |
| --- | --- | --- | --- | --- |
| **New psychotropic medication (any)** | 1467/13,594  (10.8) | 13,312/288,222  (4.6) | 2.42  (2.29 – 2.55) | 1.38  (1.30 – 1.46) |
| **New antidepressant** | 1310/15,236  (8.6) | 11,344/298,094  (3.8) | 2.33  (2.20 – 2.47) | 1.47  (1.38 – 1.56) |
| **New anxiolytic or hypnotic** | 1060/17,890  (5.9) | 8365/326,735  (2.6) | 2.36  (2.22 – 2.52) | 1.35  (1.26 – 1.44) |
| **New antipsychotic or mania medication** | 307/20,801  (1.5) | 2235/343,178  (0.7) | 2.29  (2.03 – 2.58) | 1.18  (1.04 – 1.34) |

^a^ Patients were only included in the denominator if they had not have received a community prescription for the specific medication category within 180 days prior to index hospitalisation *and* 90 days after index discharge.

^b^ Adjusted for sex, age group, ethnicity, Scottish Index of Multiple Deprivation (SIMD) quintile, main condition at hospital admission, index morbidity category (five years prior to hospitalisation), previous psychiatric admission category (five years prior to hospitalisation), previous hospital admission category (one year prior to hospitalisation), and previous emergency attendances category (one year prior to hospitalisation). *CI:* confidence interval; *HR:* hazard ratio

### Supplementary Table S6. Effect modification analyses for the association between critical care hospitalisation and new psychotropic prescription within 90 days of hospital discharge in hospital survivors.

|  |  | **Number of new prescription/naïve critical care survivors^a^ (%)** | **Number of new prescription/naïve non-critical care survivors^a^ (%)** | **Adjusted HR^b^**  **(95% CI)** | **p-value for interaction** |
| --- | --- | --- | --- | --- | --- |
| Overall |  | 1610/15,609 (10.3%) | 9743/307,429 (3.2%) | 2.03 (1.91 – 2.16) |  |
| Age | <60 years  60+ years | 704/6532 (10.8)  906/9077 (10.0) | 5502/166,911 (3.3)  4241/140,518 (3.0) | 2.02 (1.86 – 2.20)  2.01 (1.86 – 2.17) | 0.934 |
| Sex | Male  Female | 990/9685 (10.2)  620/5924 (10.4) | 4266/153,280 (2.8)  5477/154,149 (3.6) | 2.28 (2.12 – 2.45)  1.74 (1.60 – 1.90) | <0.001 |
| SIMD quintiles | 1  2  3  4  5 | 275/2242 (12.3)  416/3664 (11.4)  252/2814 (9.0)  253/2745 (9.2)  403/3979 (10.1) | 1558/44,552 (3.5)  2434/72,925 (3.3)  1827/54,972 (3.3)  1587/54,713 (2.9)  2225/76,383 (2.9) | 2.11 (1.85 – 2.40)  2.03 (1.82 – 2.25)  1.65 (1.44 – 1.88)  2.03 (1.77 – 2.32)  2.27 (2.04 – 2.53) | <0.001 |
| Comorbidities | 0  1  2+ | 787/8136 (9.7)  283/2665 (10.6)  540/4808 (11.2) | 6523/269,314 (2.4)  1468/19,227 (7.6)  1752/18,888 (9.3) | 3.75 (3.47 – 4.05)  1.34 (1.17 – 1.52)  1.24 (1.12 – 1.36) | <0.001 |

^a^ Patients were only included in the denominator if they had not have received a community prescription for the specific medication category within 180 days prior to index hospitalisation.

^b^ Adjusted for sex, age group, ethnicity, Scottish Index of Multiple Deprivation (SIMD) quintile, main condition at hospital admission, index morbidity category (five years prior to hospitalisation), previous psychiatric admission category (five years prior to hospitalisation), previous hospital admission category (one year prior to hospitalisation), and previous emergency attendances category (one year prior to hospitalisation).

*HR:* hazard ratio; *CI:* confidence interval; *SIMD*: Scottish Index of Multiple Deprivation

### Supplementary Table S7. Sensitivity analyses for the association between critical care hospitalisation and new psychotropic prescription within 90 days of hospital discharge, *censoring for hospital readmission*.

| **Psychotropic medication class** | **Number of new prescription/naïve critical care survivors^a^**  **(%)** | **Number of new prescription/naïve non-critical care survivors^a^**  **(%)** | **Unadjusted HR (95% CI)** | **Adjusted HR^b^ (95% CI)** |
| --- | --- | --- | --- | --- |
| **New psychotropic medication (any)** | 1309/15,609  (8.4) | 8692/307,429  (2.8) | 3.29  (3.10 – 3.48) | 2.11  (1.98 – 2.25) |
| **New antidepressant** | 854/17,207  (5.0) | 6537/317,708  (2.1) | 2.66  (2.48 – 2.86) | 1.90  (1.76 – 2.06) |
| **New anxiolytic or hypnotic** | 1063/20,108  (5.3) | 5412/346,609  (1.6) | 3.71  (3.48 – 3.97) | 2.33  (2.16 – 2.51) |
| **New antipsychotic or mania medication** | 181/22,412  (0.8) | 1491/361,012  (0.4) | 2.11  (1.81 – 2.46) | 1.24  (1.05 – 1.47) |

^a^ Patients were only included in the denominator if they had not have received a community prescription for the specific medication category within 180 days prior to index hospitalisation.

^b^ Adjusted for sex, age group, ethnicity, Scottish Index of Multiple Deprivation (SIMD) quintile, main condition at hospital admission, index morbidity category (five years prior to hospitalisation), previous psychiatric admission category (five years prior to hospitalisation), previous hospital admission category (one year prior to hospitalisation), and previous emergency attendances category (one year prior to hospitalisation).

*CI:* confidence interval; *HR:* hazard ratio

### Supplementary Figure S1. Hospitalised survivors prescribed psychotropic medications within 90 days after critical care (n=7527, left) and non-critical care hospitalisation (n=54,589, right).


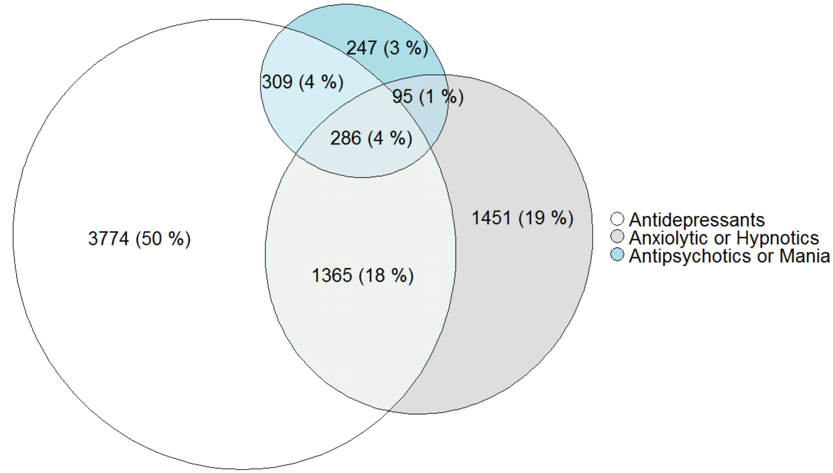

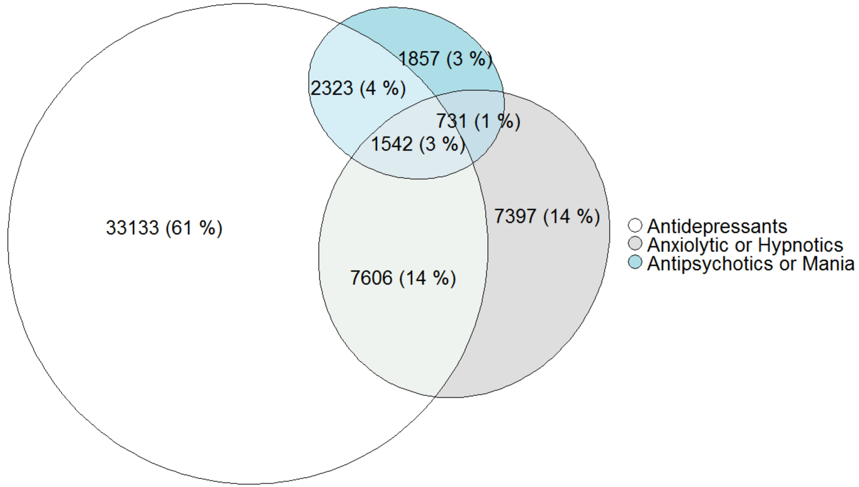


### Supplementary Figure S2. Psychotropic-naïve^a^ hospitalised survivors prescribed psychotropic medications within 90 days after critical care (n=1610, left) and non-critical care hospitalisation (n=9743, right).


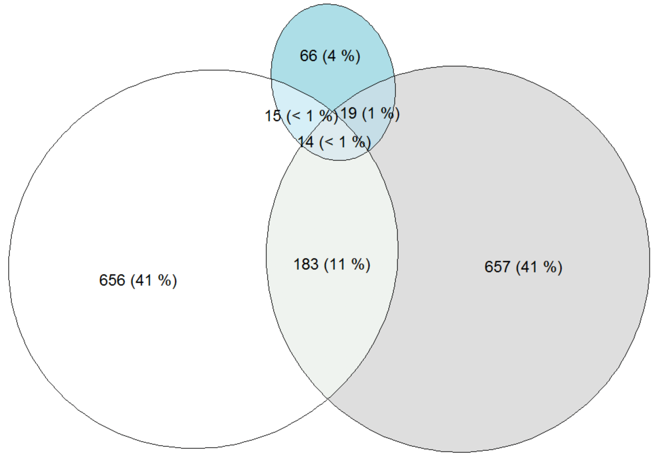

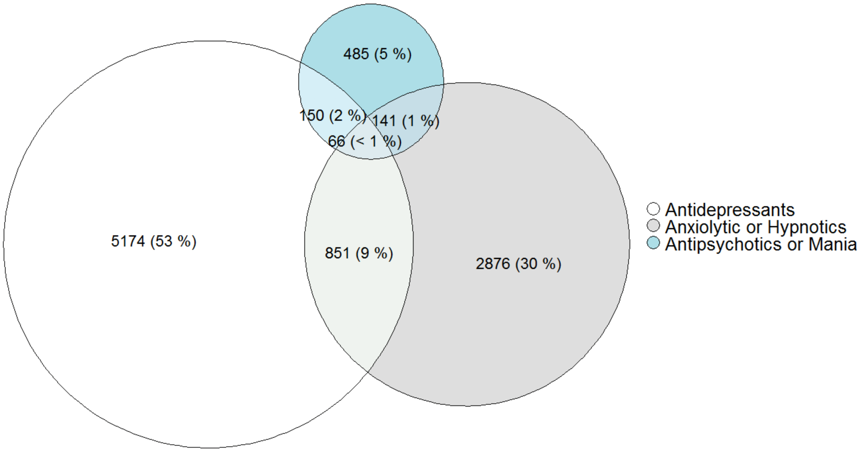


*^a^ Psychotropic-naïve patients are those that did not receive a community prescription for a psychotropic within 180 days prior to index hospitalisation.*

### Supplementary Figure S3. Cumulative incidence of new (A) antidepressant; (B) anxiolytic or hypnotic; and (C) antipsychotic or mania medication prescription within 90 days of hospital discharge in critical care vs non-critical care medication-naïve^a^ patients.

(A)

(B)

(C)

^a^ Patients were only included in the denominators if they had not have received a community prescription for the specific medication category within 180 days prior to index hospitalisation.
